# Comparison of Dental Zirconium Oxide Ceramics Produced Using Additive and Removal Technology for Prosthodontics and Restorative Dentistry—Strength and Surface Tests: An In Vitro Study

**DOI:** 10.3390/ma17010168

**Published:** 2023-12-28

**Authors:** Wojciech Frąckiewicz, Marcin Królikowski, Konrad Kwiatkowski, Ewa Sobolewska, Paweł Szymlet, Małgorzata Tomasik

**Affiliations:** 1 Department of Dental Prosthetics, Faculty of Medicine and Dentistry, Pomeranian Medical University in Szczecin, 70-111 Szczecin, Poland; 2 Department of Manufacturing Engineering, Faculty of Mechanical Engineering and Mechatronics, West Pomeranian University of Technology in Szczecin, 70-310 Szczecin, Poland; 3 Department of Mechanics and Fundamentals of Machine Design, Faculty of Mechanical Engineering and Mechatronics, West Pomeranian University of Technology in Szczecin, 70-310 Szczecin, Poland; 4 Department of Interdisciplinary Dentistry, Faculty of Medicine and Dentistry, Pomeranian Medical University in Szczecin, 70-111 Szczecin, Poland

**Keywords:** 3D, three-dimensional, additive-manufacturing, zirconium, zirconium paste, 3D printing, dentistry, prosthodontics, 3DCeram, Ivoclar Digital

## Abstract

Background: The aim of this in vitro study was to determine the mechanical and functional properties of zirconium oxide ceramics made using 3D printing technology and ceramics produced using conventional dental milling machines. Methods: Forty zirconia samples were prepared for this study: the control group consisted of 20 samples made using milling technology, and the test group consisted of 20 samples made using 3D printing technology. Their surface parameters were measured, and then their mechanical parameters were checked and compared. Density, hardness, flexural strength and compressive strength were tested by performing appropriate in vitro tests. After the strength tests, a comparative analysis of the geometric structure of the surfaces of both materials was performed again. Student’s *t*-test was used to evaluate the results (*p* < 0.01). Results: Both ceramics show comparable values of mechanical parameters, and the differences are not statistically significant. The geometric structure of the sample surfaces looks very similar. Only minor changes in the structure near the crack were observed in the AM group. Conclusion: Ceramics made using additive technology have similar mechanical and surface parameters to milled zirconium oxide, which is one of the arguments for the introduction of this material into clinical practice. This in vitro study has shown that this ceramic can compete with zirconium made using CAD/CAM (Computer-Aided Design and Computer-Aided Manufacturing) methods.

## 1. Introduction

Prosthetic restorations in dentistry can be divided into removable and fixed. The most popular removable restorations include partial or complete dentures, most often made of materials such as alloys of various metals, acrylic or more modern materials such as nylon, acetal or polyether ether ketone (PEEK), which were tested in recent studies [1,2]. Dental ceramics are often used to make permanent restorations placed on the patient’s own teeth or implanted abutments. All-ceramic restorations have different mechanical and aesthetic properties, depending on the type of material used and the technology of their production [3,4]. Fixed restorations made on a metal base and then fused with feldspathic porcelain also have appropriate strength parameters but are much less aesthetic, mainly due to exposing the metal beneath in the cervical region of the tooth [5]. Zirconium oxide is a material characterized by high mechanical strength, crack resistance, high biocompatibility and good aesthetic effect. The features of this material allow it to be often used in dentistry for making small permanent restorations such as inlays, onlays or overlays, as well as crowns and extensive bridges based on teeth or dental implants [6,7,8].

In dentistry, zirconium oxide began to be used in the 1970s. The combination of high aesthetics and functionality means that the indications for its use are constantly expanding [9]. Currently, zirconium oxide prosthetic restorations can be made using several technologies. The most popular and widely used is the subtractive method, called CAD/CAM technology which involves milling a permanent prosthetic restoration designed in special software using a milling machine [10,11,12]. Currently, there are many systems for producing zirconium oxide restorations on the market, and the most famous ones include Lava (3M-ESPE, Neuss, Germany), Procera (NobelBiocare, Mahwah, NJ, USA), Cercon (Dentsply Sirona, South Carolina, USA), Everest (KaVo Dental, Biberach an der riß, Germany) and IPS e.max (Ivoclar Digital, Schaan, Liechtenstein). 

The development of digitalization has allowed for the introduction of 3D printing into dentistry, which enables faster performance of individual stages of treatment in the dental office. This technology is used, among others, in surgery—thanks to the use of printed templates for inserting implants [13,14], as well as in orthodontics, allowing for the printing of special overlays or brackets for teeth using software adapted for this purpose [15,16]. This technology is also used in dental prosthetics. Three-dimensional printers can work with intraoral scanners and CAD/CAM softwares, which reduces the risk of human error and ensures the repeatability of results [17]. Based on a scan of a patient’s mouth, a model of his teeth can be printed, avoiding the standard procedure of taking a conventional impression and casting a model of the teeth from an appropriate plaster. The use of a printer that operates unattended most of the time reduces the workload of the dental technician and enables the automation of the entire process [18,19]. 

The dynamic development of 3D printing has also allowed for the production of prosthetic restorations made of zirconium oxide using this technology. Most often, a variety of zirconium oxide stabilized with yttrium is used for this type of print [20]. Currently, several manufacturers offer various technologies that enable the production of prosthetic restorations by printing with zirconium oxide. A popular material is Lithoz 230Y (Lithoz, Vienna, Austria), produced using the proprietary LCM (Lithography-based Ceramic Manufacturing) technology of Lithoz (Vienna, Austria), in which the ceramic suspension is hardened layer-by-layer in visible (blue) light [21]. Another material used is 3DMix ZrO2 (3DCeram, Bonnac-la-Côte, France), intended for printing using stereolithography (SLA) technology, in which a computer-controlled laser beam illuminates the surface of a photosensitive resin in the dictated shape of a given object, causing its polymerization [22]. SLA technology differs from DLP (Digital Light Processing) technology in that in SLA, the resin is hardened one point at a time, while in DLP, the entire layer of resin is hardened simultaneously [23].

In the case of zirconium oxide made using the subtractive technique, many authors point out [24,25] its high resistance to cracking and compression and also present the geometric structure of its surface [26]. In publications describing the mechanical properties and surface geometry characteristics of materials made using additive technology [27,28], there is insufficient data on density or hardness, as well as a very important feature for dental materials, which is their resistance to compression. Some authors have shown that the flexural strength of printed zirconia is lower than that of milled zirconia [29], but there are very few research results relating to the compressive strength of these materials.

The aim of this research was to compare the mechanical properties and assess the impact of strength tests on the change in the geometric structure of the surface of zirconium oxide produced by milling and 3D printing. The null hypothesis assumed that the tested materials have the same mechanical properties.

## 2. Materials and Methods

**Figure 1:**
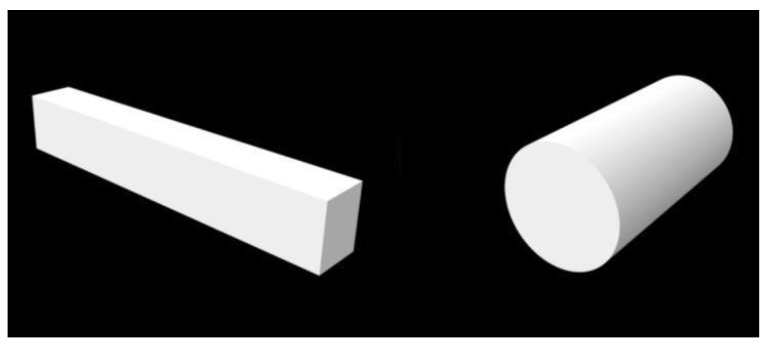
Schematic of the samples made using an STL file.

Twenty bars made of yttrium-stabilized zirconium oxide with the dimensions of 30 × 5 × 4 mm and 20 cylinders with the dimensions of φ5 × 10 mm [30] were made with two technologies: 10 samples of a given shape using subtractive technology (SM group) and additive technology (AM group). The samples were designed using special software (Exocad Rijeka 3.1, Exocad GmbH, Darmstadt, Germany), and their sample shape models (Figure 1) were exported in STL format.

Samples from the SM group (*n* = 20) were made of monolithic discs of yttria-stabilized zirconium oxide (IPS e.max ZirCAD LT, Ivoclar Digital, Schaan, Liechtenstein) in shade A2. The samples were milled on a 4-axis milling and grinding machine (Z4, VHF, Ammerbuch, Germany), and then, in the device program (DentalCAM 7.08, VHF, Ammerbuch, Germany), a pattern was programmed on the discs for milling the samples with a magnification of 1.218 because zirconium oxide reduces its size during sintering. The samples were then sintered in an oven (HT-S Speed, Mihm-Vogt, Stutensee, Germany) to a temperature of 1530 degrees Celsius and then cooled slowly to room temperature according to the manufacturer’s recommendations [31].

Samples in the AM group (*n* = 20) were made using a 3D printer (Ceramaker C900, 3DCeram, Bonnac-la-Côte, France), in which polymerization took place using a UV laser with a length of 355nm and a sintering point diameter of ~35 μm located at the base of the platform. This printer prints using SLA (stereolithographic) technology, based on the suspension photopolymerization process. The slurry used to print parts (3DMix ZR3-F01, 3DCeram, Bonnac-la-Côte, France) is composed of ceramic powder (medical-grade zirconium oxide) and a photosensitive polymer. This polymer, or organic substances, was burned during the debonding process. Printed samples were heated gradually in an oven (HTL 20/17, Thermconcept GmbH, Bremen, Germany) to a temperature of 1450 degrees Celsius and then cooled to room temperature according to the manufacturer’s recommendations. After sintering the suspension, only the ceramic structure remained [32].

### 2.1. Assessment of Flexural Strength

**Figure 2:**
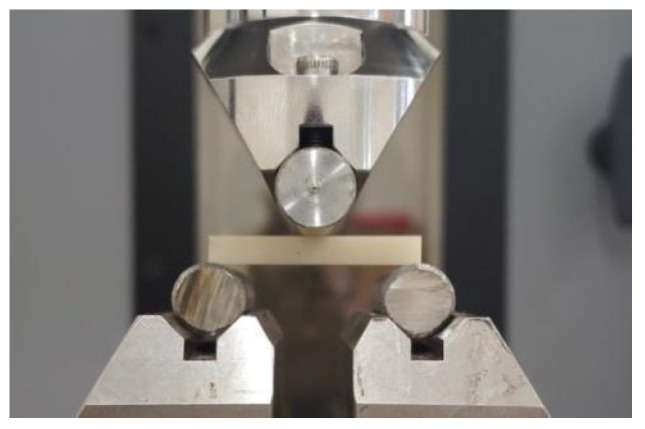
Specimen during bending test.

A bending test was performed on a three-point system consisting of two cylindrical supports with a diameter of 10 mm placed at a distance of L = 28 mm. Ten bar-shaped samples from each group were used for the test (*n* = 10), and the measurements were performed using a universal testing machine (ElectroPuls E10000, Instron, Norwood, MA, USA). The application point of the cylindrical load cell was located in the center of the sample (Figure 2), and the loading speed was 1 mm/min. During the test, force and displacement were recorded and converted into relative stresses and elongations. The test was carried out until the sample cracked and the maximum stress that the material could withstand was determined. The stress–strain characteristics corrected for the stiffness of the system allowed for the determination of longitudinal elasticity coefficients (Young’s modulus) for the tested materials.

### 2.2. Assessment of Compressive Strength

**Figure 3:**
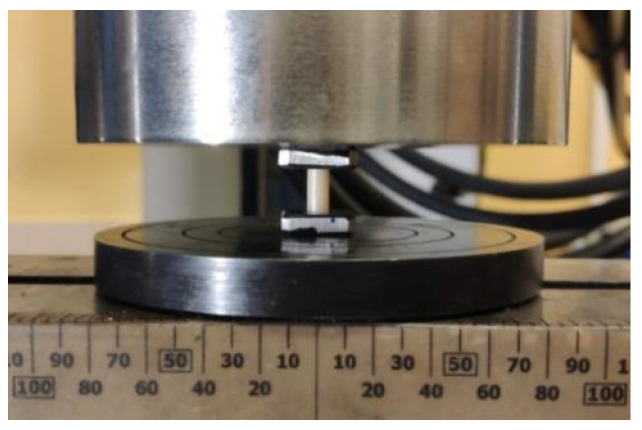
Arrangement of the system for measuring compressive strength.

The compressive strength of zirconium ceramics from both groups was measured. For the test, 10 cylinder-shaped samples from each group (*n* = 10) were used, which were placed perpendicular to the force of the device. Measurements were made using a universal servo-hydraulic testing machine (Instron 8850, Instron, Norwood, MA, USA). The deformation speed was 0.5 mm/min. Due to the very high stresses acting on the sample bases during compression, which could plastically indent the compression tables and damage them, the samples were compressed between sintered carbide plates (Figure 3). The maximum stress that the material can carry during compression to failure was determined. The stress–strain characteristics corrected for the stiffness of the system were also determined, and Young’s modulus in compression was determined.

### 2.3. Density Assessment

**Figure 4:**
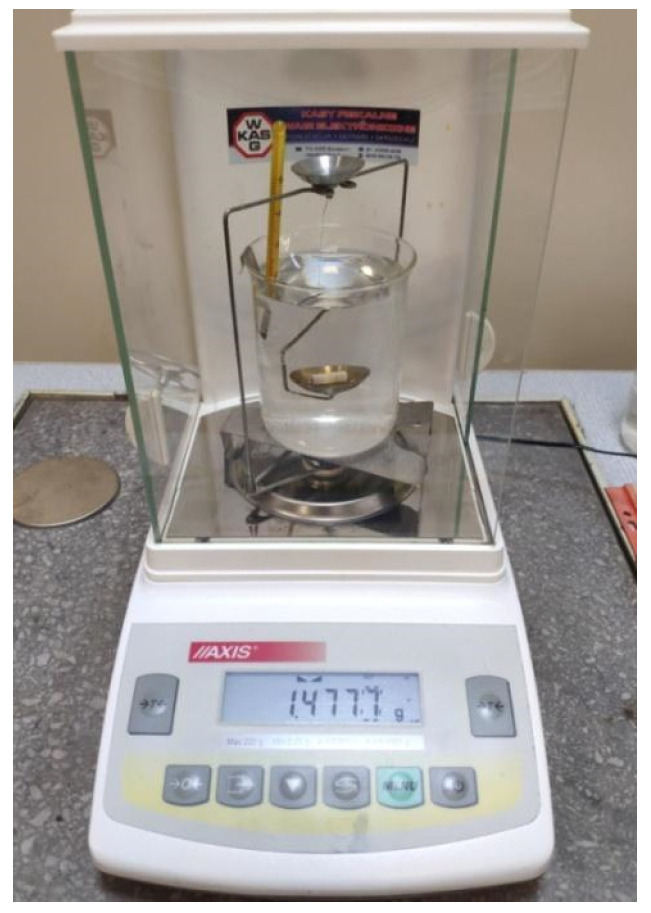
Density measurement system.

Density was measured using a laboratory hydrostatic balance (ALZ60, AXIS, Gdańsk, Poland), which contains an appropriate kit for determining density with an accuracy of 0.015 g/cm^3^ (Figure 4), in accordance with the ISO 1183-1 standard [33]. Five samples from each group were used for measurements (*n* = 5). The measurement was made at a temperature of 23 °C using the following formula:

$$d=\frac{m_{a}}{\left(m_{a\;}-m_{w\;}\right)}\;\cdot \;d_{w\;}+d_{a}$$

where *m_a_* is the mass of the sample in air, *m_w_* is the mass of the sample in water, *d_w_* is the density of water at 23 °C and *d_a_* is the density of air.

### 2.4. Hardness Testing

**Figure 5:**
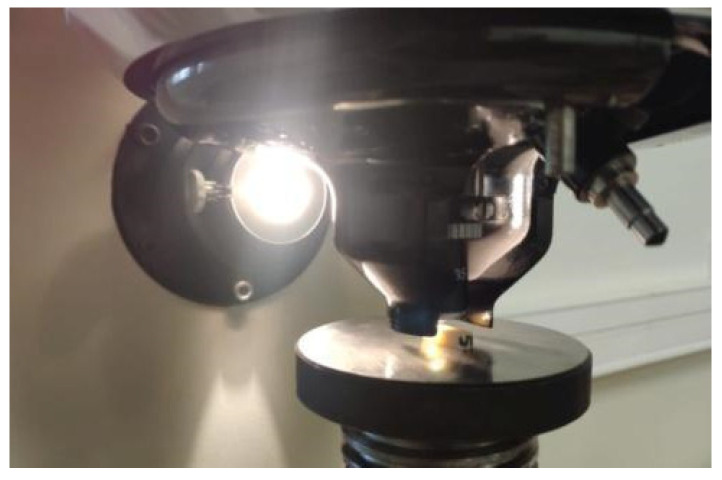
Station for measuring Vickers hardness.

Vickers hardness measurements were performed for each sample in two different places (*n* = 20). The measurements were made using a universal hardness tester (Wilson UH930, Buehler, Esslingen, Germany) by indenting with a force of 490.4 N for 10 s (HV50)—Figure 5. Then, the diagonals of the resulting impression were measured and, after entering the data into the system, the hardness for a given sample was calculated based on the area of the resulting cross-section.

### 2.5. Analysis of the Geometric Structure of the Surface

To test the geometric structure, 10 bar-shaped samples from both groups were used, 5 from each group (*n* = 5). After production, the samples were not subjected to any processing to see what their surface looked like immediately after the sintering process. For the samples made using the subtractive method, surface parameters were checked on smooth surfaces, avoiding the surfaces where the samples were in contact with the disc holding them in the milling machine holder. For the 3D printing samples, this was not necessary because the surfaces were smooth from the beginning. Before the strength tests, the first measurement was made and then repeated on the bars cracked in half after the three-point bending test.

#### 2.5.1. Roughness Assessment

**Figure 6:**
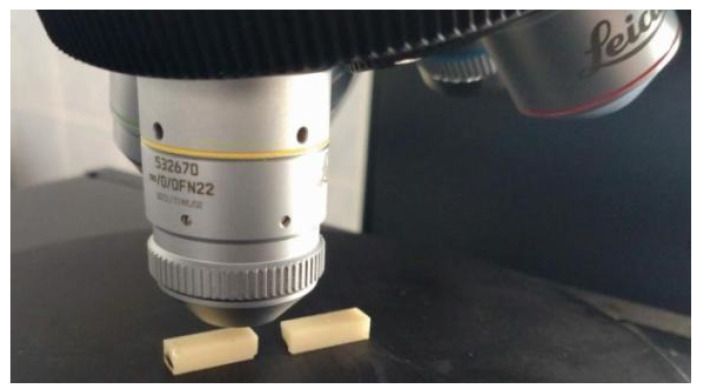
Geometric structure of the surface measurement of the sample surface near the fracture.

A 3D Surface Metrology Microscope (DCM8, Leica Microsystems, Wetzlar, Germany) was used to assess the geometric structure of the sample surfaces. The microscope is equipped with an anti-vibration base, a rotating stage and a turret with objectives (Figure 6). The microscope can operate in profilometer and optical microscopy mode. The available operating modes are confocal, interferometric and variable focus. The microscope works with lighting of any color configuration.

Bar-shaped samples were measured at 5 points from both the AM (*n* = 5) and SM (*n* = 5) groups. In order to avoid reflections resulting from the external structure of the samples, the interferometric mode was used. In accordance with the ISO 25178 standard [34], the parameters presented in Table 1 were estimated.

Measurements in the interferometric mode were performed using a dedicated objective (HC PL FLUOTAR 10x/0.30 3.57 Mirau, Leica Microsystems, Wetzlar, Germany).

#### 2.5.2. Visual Assessment of Crack Propagation

**Figure 7:**
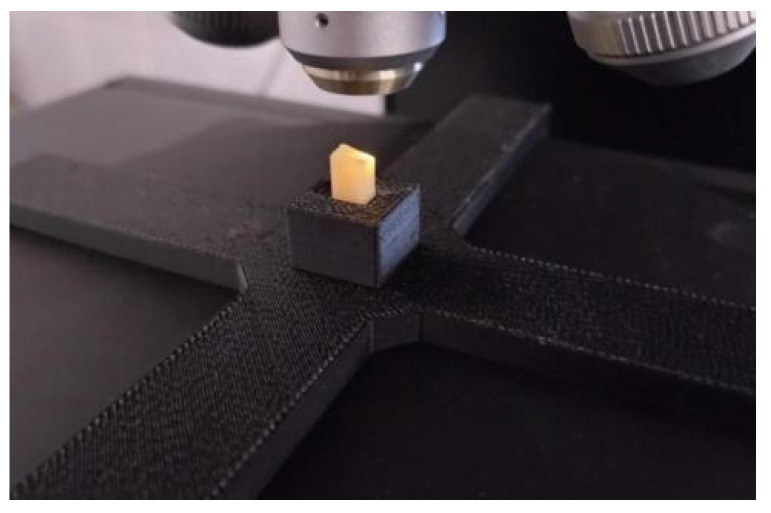
Imaging of the sample surface in a fracture. The handle was printed with a 3D printer for repeatability.

In order to visually analyze the propagation and form of cracks inside the material, fracture measurement was performed for selected samples after milling and sintering (AM). The forms of breakthroughs did not differ very much. However, the fractures of samples made using the two compared methods were so irregular that no numerical parameters were collected for them, e.g., Sv, Sa, or Sq. Imaging of breakthroughs was performed in variable focus mode with a dedicated lens (N PLAN L 20X 0.40 10.8, Leica Microsystems, Wetzlar, Germany) using white light (Figure 7).

### 2.6. Statistical Analysis

For descriptive analysis, the mean and standard deviation of a given group of ceramics were calculated. Student’s *t*-test was used to assess the normality of data distribution (*p* < 0.01). The data were distributed normally. A comparison of the mean results of both materials was made based on the ISO 2854 standard [35], and the results for each feature are presented in Table 2 with a confidence interval of 99%.

## 3. Results

### 3.1. Assessment of Flexural Strength

The load at break was recorded for each sample and their flexural strength was calculated. In the case of the SM group, it was 688 ± 100 MPa (99% confidence interval [CI] 588 to 788 MPa), and in the AM group, it was 813 ± 265 MPa (99% CI 548 to 1078 MPa). The arithmetic means of both materials did not differ statistically at the given test significance level (*p* < 0.01). Flexural Young’s moduli were also recorded for both materials, which were 203 ± 11 GPa (99% CI 192 to 214 GPa) for the AM group and 206 ± 11 GPa (99% CI 195 to 217 GPa) for the SM group, respectively. Using the same statistical method as for the assessment of bending strength, no statistically significant differences were found (*p* < 0.01).

### 3.2. Assessment of Compressive Strength

Cylindrical samples were loaded to failure, and their compressive strength was determined. In the SM group, the compressive strength was 3.99 ± 0.65 GPa (99% CI 3.34 to 4.64 GPa), and in the AM group, it was 4.16 ± 0.53 GPa (99% CI 3.63 to 4.69 GPa). In the statistical analysis at the test significance level (*p* < 0.01), the compressive strength results of the tested materials did not differ from each other. The Young’s moduli in compression were 231 ± 37 GPa (99% CI 194 to 268 GPa) for the AM group and 230 ± 31 GPa (99% CI 199 to 261 GPa) for the SM group, respectively, and no statistical differences were found between the materials at the significance *p* < 0.01.

### 3.3. Density Assessment

Five samples were selected for density measurement from each group. In the SM group, the average density was 6.056 ± 0.031 g/cm^3^ (99% CI 6.025 to 6.087 g/cm^3^), and in the AM group, it was 6.003 ± 0.035 g/cm^3^ (99% CI 5.968 to 6.038 g/cm^3^). Static analysis at the significance level of *p* < 0.01 indicated that the differences in material densities were statistically significant.

### 3.4. Hardness Testing

The SM group showed lower hardness values than the AM group at 1285 ± 30 HV (99% CI 1255 to 1315 HV) and 1319 ± 23 HV (99% CI 1296 to 1342 HV), respectively. The estimated means did not differ from each other statistically at the significance level of *p* < 0.01. All mechanical parameter results are compared in Table 2.

### 3.5. Analysis of the Geometric Structure of the Surface

#### 3.5.1. Assessment of the Geometric Structure of Surfaces

Measurements were made on samples with a rectangular cross-section both before and after the strength test. Before the test, each sample was measured at the center where the breakthrough was expected. After the test, measurements were taken in two places immediately around the breakthrough. 

**Figure 8:**
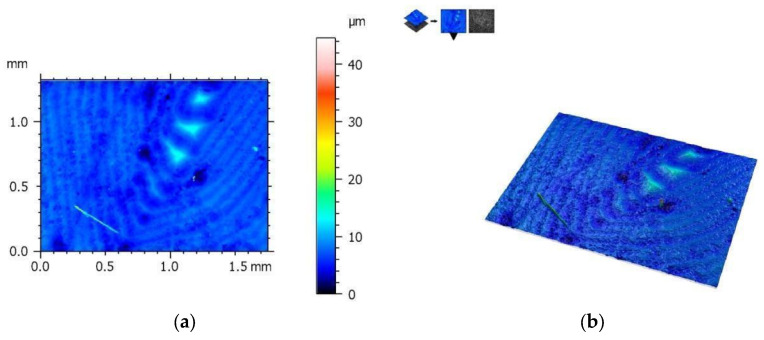
View of the height histogram in simulated colors for one of the samples from the SM group: (**a**) 2D view and (**b**) 3D view.

**Figure 9:**
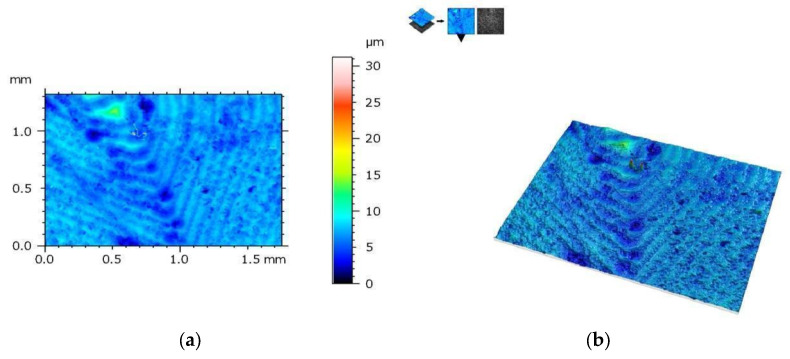
The same sample after fracture (SM group)—geometric structure of the surface: (**a**) 2D view and (**b**) 3D view.

The measurement protocol with the collected geometric structure of the surface data from a surface section of 1753 (X) × 1320 mm (Y) with a resolution of 1360 (X) × 1024 points (Y) is presented in the figures below. The geometric structure of the surface measurement results of samples from the SM group before fracture are shown in Figure 8, while Figure 9 shows the surface of the same sample after the bending test.

**Figure 10:**
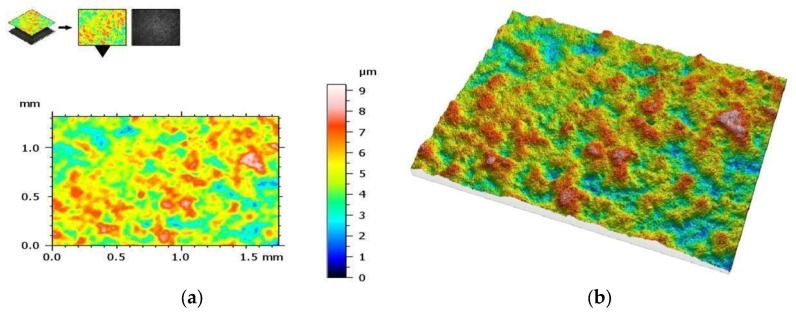
View of the height histogram in simulated colors for one of the samples from the AM group: (**a**) 2D view and (**b**) 3D view.

**Figure 11:**
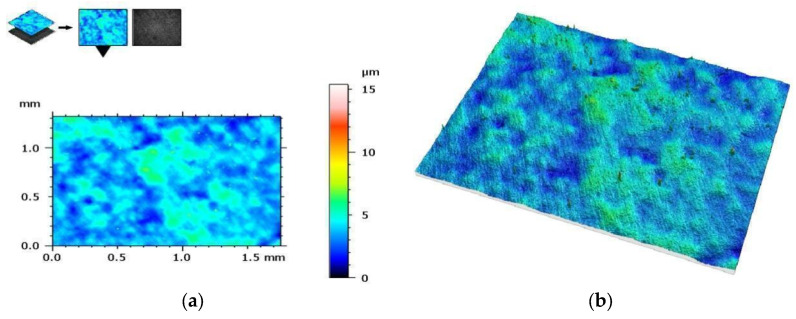
The same sample after fracture (AM group)—geometric structure of the surface: (**a**) 2D view and (**b**) 3D view.

On the other hand, Figure 10 shows the measurement results of the AM group samples before bending tests, and Figure 11 shows the measurement results after these tests. The results are presented from the center of the specimen, where fracture is expected in the three-point bending test. 

Table 3 and Table 4 summarize the results of the geometric structure of the surface parameters for both groups. The tests showed that the samples made additively (AM group) and subtractively (SM group) have only minor changes near the crack, but in the case of the samples from the AM group, these changes are minimal. When examining the crack form under a microscope, noticeable similarities are observed between both types of materials.

#### 3.5.2. Visual Assessment of Crack Propagation

**Figure 12:**
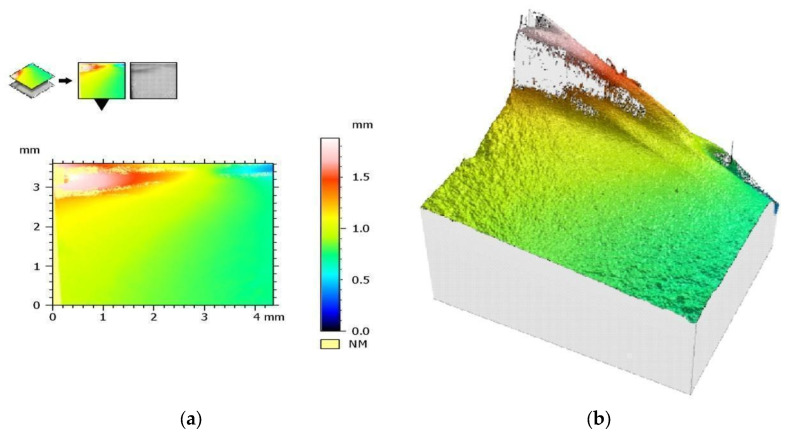
Sample from the SM group: (**a**) a color histogram of simulated breakthrough forms and (**b**) a 3D color view of simulated fracture forms.

**Figure 13:**
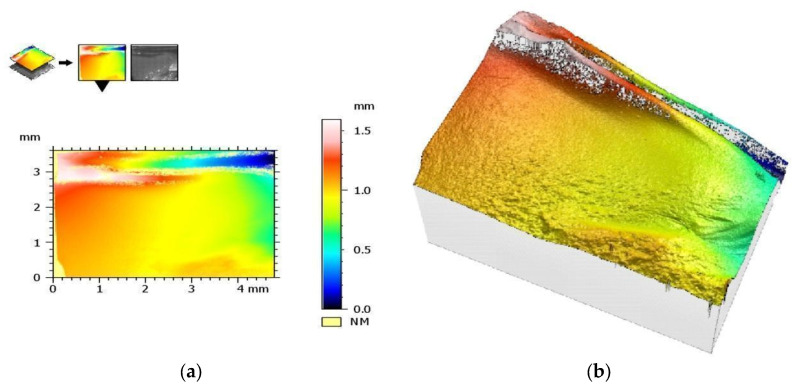
Sample from the AM group: (**a**) a color histogram of simulated breakthrough forms and (**b**) a 3D color view of simulated fracture forms.

A visual assessment of the crack shape for samples from the SM group is presented in Figure 12, and a similar assessment for a sample from the AM group is presented in Figure 13. The crack shape is very similar.

## 4. Discussion

In modern dentistry, a big challenge is to create a durable and biocompatible prosthetic restoration. The expectations of clinicians and the needs of patients are met using materials with properties similar to human tooth tissues, including zirconium oxide as described in this study. The development of the latest technologies allows for the use of various methods to produce this material. The above in vitro study was aimed at comparing mechanical parameters, such as density and hardness, flexural and compressive strength, and the geometric structure of milled and additively manufactured zirconium oxide.

The originally assumed null hypothesis was fully confirmed with the endurance tests. The tests showed no statistically significant differences between the two materials at the 0.01 significance level. Our study did not show a statistical difference between the materials, contrary to the study by Baysal et al. [36], where printed zirconia showed weaker results than milled zirconium oxide with a value of 1501 ± 60 HV in the SM group, i.e., a significantly higher HV value than in the AM group (1169 ± 48 HV). The reason for the difference in both studies may be the samples used in both studies: despite using the same study material, Baysal et al.’s samples were made with another printer (Carmel 1400, XJet, Rehovot, Israel). It would take more research to show the differences between these two printers.

The flexural strength of natural molars is between 103 and 167 MPa, so regardless of the test results of the AM group or SM group, it is sufficient to replace natural teeth. The flexural strength in our own tests was 813 ± 265 MPa for the AM group and 688 ± 100 MPa for the SM group. The difference, although quite significant, is not statistically significant at the 0.01 level of significance due to the large scatter of partial results, which is typical for brittle materials. Revilla-León et al. [29] used the same material to test this feature, but the result of their research indicated completely opposite results: 320 ± 40 MPa and 914 ± 68 MPa for the AM and SM groups, respectively. On the other hand, the results of own research agree with the results of the work of Zandinejad et al. [37], who calculated that their AM zircon with 0% porosity (AMZ0) showed the highest flexural strength value of 755 ± 147 MPa. Their findings also disagree with those of the previous author [29]. In another study, Revilla-León et al. [38] used a material produced using LCM technology (LithaCon 3Y 210; Lithoz, Vienna, Austria) instead of zirconium produced using SLA (3DMix ZrO2, 3DCeram, Bonnac-la-Côte France) and obtained results of 1519 ± 259 MPa for 3D printing samples, which obtained significantly higher average values of bending strength compared with milled samples (981 ± 130 MPa). The reason for such visible differences between studies can be explained by different chemical compositions of the materials, changes during production procedures, or differences in the porosity of a given material. Some authors also believe that the post-production process of zirconium may affect its mechanical properties, such as its polishing, which can significantly improve its bending strength [39].

The current literature lacks information on the compressive strength of printed zirconia, even though it is a very important feature of a material used in the oral cavity. Our own tests showed compressive strength of 3.99 ± 0.65 GPa in the SM group and 4.16 ± 0.53 GPa in the AM group. Among the articles, only one paper [28] discussed additively produced zirconium oxide with respect to this feature, but due to the different material and production process, as well as very low results of 60–172 MPa, which seem unreliable, there is no reason to compare the results of these studies.

Even though Young’s modulus is a material constant, it is typical that higher Young’s modulus results are obtained in compression (or tension) than in bending. This is due to, among others, the failure to take into account shear forces during analytical calculations for bending and the fact that in bending, the stress distribution is very uneven compared with compression. The elasticity of both tested ceramics, both in bending and in compression, as expressed by the calculated Young’s modulus, turned out to be so similar for both materials that the authors decided not to calculate the Poisson’s and Weibull’s ratio. 

Density is one of the characteristics of ceramics that can have a large impact on its mechanical properties. In the study by Opalinska et al. [40], it was found that as the size of nanoparticles decreases or the specific surface area increases, the density of nanoparticles decreases [22]. This may be due to the production technique or the quality of the material. The authors of the studies on zirconium oxide [36,41] obtained density results of ∼6 g/cm^3^. In our own research, very similar results were obtained: in the AM group, a lower ceramic density was found than in the SM group with values of 6.003 ± 0.035 g/cm^3^ and 6.056 ± 0.031 g/cm^3^, respectively. These differences, although small, are statistically significant at the significance level of *p* < 0.01.

SLA zirconium oxide exhibits different mechanical properties depending on the roughness of its surface. Arranging the printed layers parallel to the stretching side improves its mechanical properties. To obtain the best quality of the material, it is necessary to reduce the surface roughness of the final product and also reduce its porosity [37]. The information resulting from these studies means that in order to obtain the best possible results for this material in future studies, its density should be high, the length of the printing layer should be as long as possible, and it should be subjected to surface treatment after sintering. Tooth restoration using additively manufactured zirconium oxide reduces the requirements for the post-production process due to the lack of a connector between the milled part and the disc or block in which it is originally held, which occurs using CAD/CAM technology [42].

Thanks to the ability to create complex designs, save materials, and produce structures not only from zirconium but also from other materials, additive manufacturing technologies may pose strong competition to traditional milled ceramics techniques in the future. Moreover, this technique allows for the creation of both partially sintered (porous) and fully sintered (solid) structures. The results of this study indicate that zirconium can be used for dental applications due to its mechanical parameters and geometric structure.

### The Limitations of This Study

The purpose of this study was to demonstrate the difference between the materials in terms of mechanical and surface parameters. Three-dimensional ceramics printing is a new technology; therefore, prints also involve high prices for the production of samples both for research purposes and initially for use in the dental industry for use on patients. Currently, there are also no clinical tests that allow for predicting how this ceramic will behave in the patient’s oral cavity because the material used for SLA printing in this study, despite containing medical-grade powdered zirconium, does not have an ISO 13485 [43] license for its use in the necessary medical industry. This is another issue that must be resolved before the material can be introduced into the broader dental market. Additionally, this study performed by the authors was not able to determine how the material behaves directly in the oral cavity. As previously performed for composite materials, the effect of immersion in acidic drinks [44] and aging [45] should also be tested by comparing both additive and removal technology for zirconium oxide ceramics.

## 5. Conclusions

This study may provide a technical justification for developing indications for the wider use of 3D-printed ceramics in clinical practice. The tests and analyses performed showed that at the 99% confidence level (*p* < 0.01), the mechanical properties of ceramics produced with additive technology are statistically the same as those of milled zirconium ceramics. The tested ceramics differ slightly in density, and in the additive technique, a material with a lower density is obtained, but the difference in density is so small that it does not affect the mechanical properties. The geometric structure of the surface analysis of samples from both the subtractively (SM) and the additively manufactured groups show only slight changes near the fracture for samples from the AM group. However, these changes are negligible. Visual microscopic assessment of the fracture form shows significant similarities for both types of samples. In terms of the tests performed, the material is able to compete with zirconium oxide made with milling technology, but in vivo tests must first be completed before it can be introduced into everyday clinical use in dentistry.

## Figures and Tables

**Table 1:** Surface parameters estimated in this study.

Sq	Root-mean-square height	$Sq=\sqrt{\frac{1}{A}\int_{}^{}\int_{A}^{}z^{2}(x,y)dxdy}{}^{}$
Ssk	Skewness	Skewness of the height distribution. $Ssk=\frac{1}{{Sq}^{3}}\left[\frac{1}{A}\int_{}^{}\int_{A}^{}z^{3}(x,y)dxdy\right]$
Sku	Kurtosis	Kurtosis of the height distribution. $Sku=\frac{1}{{Sq}^{4}}\left[\frac{1}{A}\int_{}^{}\int_{A}^{}z^{4}(x,y)dxdy\right]$
Sp	Maximum peak height	Height between the highest peak and the mean plane.
Sv	Maximum pit depth	Depth between the mean plane and the deepest valley.
Sz	Maximum height	Height between the highest peak and the deepest valley.
Sa	Arithmetic mean height	Mean surface roughness. $Sa=\frac{1}{A}\int_{A}^{}\left|z(x,y)\right|dxdy$

**Table 2:** Results of measurements of mechanical parameters.

Characteristic	SM Group		AM Group	
	Mean Value	Confidence Interval 99%	Mean Value	Confidence Interval 99%
Flexural strength (MPa)	688	100	813	265
Young modulus—for flexural strength (GPa)	203	11	206	11
Compressive strength (GPa)	3.99	0.65	4.16	0.53
Young modulus—for compressive strength (GPa)	230	31	231	37
Density (g/cm^3^)	6.056	0.031	6.003	0.035
Hardness (HV)	1285	30	1319	23

**Table 3:** Geometric structure of the surface results for the SM group samples before and after rupture.

**Summary of the Geometric Structure of the Surface Parameters for Samples from the SM Group before Cracking**					
**Parameter**	**Unit**	**Mean Value**	**Standard Deviation**	**Minimum**	**Maximum**
Sq	µm	1.19	0.29	0.89	1.69
Ssk		2.5	3.6	−1.2	8.1
Sku		78	88	6	235
Sp	µm	26	16	6	44
Sv	µm	9.7	2.5	6.7	13.1
Sz	µm	35	17	14	56
Sa	µm	0.74	0.10	0.62	0.92
**Summary of the Geometric Structure of the Surface Parameters for Samples from the SM Group after Cracking**					
**Parameter**	**Unit**	**Mean Value**	**Standard Deviation**	**Minimum**	**Maximum**
Sq	µm	1.17	0.29	0.83	1.52
Ssk		2.1	4.5	−1.0	10.9
Sku		89	102	10	288
Sp	µm	32	14	13	48
Sv	µm	9.6	2.9	6.4	14.5
Sz	µm	42	16	22	63
Sa	µm	0.76	0.13	0.59	0.92

**Table 4:** Geometric structure of the surface results for the AM group samples before and after rupture.

**Summary of the Geometric Structure of the Surface Parameters for Samples from the AM Group before Cracking**					
**Parameter**	**Unit**	**Mean Value**	**Standard Deviation**	**Minimum**	**Maximum**
Sq	µm	0.99	0.36	0.34	1.39
Ssk		−0.08	0.16	−0.36	0.10
Sku		4.1	2.7	2.6	9.5
Sp	µm	5.04	0.91	3.92	6.48
Sv	µm	4.17	0.68	3.30	5.06
Sz	µm	9.2	1.1	7.2	10.3
Sa	µm	0.79	0.30	0.26	1.12
**Summary of the Geometric Structure of the Surface Parameters for Samples from the AM Group after Cracking**					
**Parameter**	**Unit**	**Mean Value**	**Standard Deviation**	**Minimum**	**Maximum**
Sq	µm	1.08	0.30	0.63	1.42
Ssk		1.9	2.4	0.0	5.8
Sku		39	52	3	138
Sp	µm	19	11	11	40
Sv	µm	6.0	2.4	3.5	10.6
Sz	µm	25	11	15	45
Sa	µm	0.82	0.25	0.42	1.07

## Data Availability

Data are contained within the article.
